# Thrombotic microangiopathy and liver toxicity due to a combination therapy of leflunomide and methotrexate: a case report

**DOI:** 10.1186/s13256-020-2349-4

**Published:** 2020-02-05

**Authors:** Rainer Ullrich Pliquett, Christoph Lübbert, Christoph Schäfer, Matthias Girndt

**Affiliations:** 1 Department of Internal Medicine 2, Halle University Hospital, Martin-Luther University Halle-Wittenberg, Ernst-Grube-Strasse 40, 06120 Halle (Saale), Germany; 2 Department of Nephrology and Diabetology, Carl-Thiem Hospital, Thiemstrasse 111, 03048 Cottbus, Germany; 3 Division of Infectious Diseases and Tropical Medicine, Department of Gastroenterology and Rheumatology, Leipzig University Hospital, Liebigstrasse 20, 04103 Leipzig, Germany

**Keywords:** Thrombotic microangiopathy, Liver toxicity, leflunomide, Methotrexate, Case report

## Abstract

**Background:**

Treatment of active rheumatoid arthritis may necessitate a methotrexate mono- or combination therapy. As in the present case, novel side effects may occur, when escalating therapy.

**Case presentation:**

A 63-year-old Caucasian female patient with rheumatoid arthritis on methotrexate for 8 years and on leflunomide for 6 years was admitted for weakness, edema, ascites, and petechiae of the lower legs. Comorbidities included a urinary tract infection, metabolic syndrome with obesity, type-2 diabetes without necessity for insulin or oral antidiabetics, and non-alcoholic fatty liver disease. Laboratory results showed acute liver failure, oliguric acute kidney injury, thrombocytopenia, and schistocyte-positive, Coombs-negative hemolytic anemia. On admission, her ADAMTS13 activity was decreased, and her leflunomide plasma level was elevated (120 μg/l). Due to severe hypoalbuminemia, an intravascular hypovolemia, and severe metabolic alcalosis with hypokalemia were found.

For the newly diagnosed thrombotic microangiopathy, leflunomide and methotrexate were discontinued, and 4 units of fresh-frozen plasma were given. Steroid therapy was administered for 5 days, until thrombotic thrombocytopenic purpura was excluded. Intravenous human albumin, oral vitamin K, and cholestyramine were administered for liver failure and leflunomide overdosage, respectively. Liver biopsy revealed a non-alcoholic fatty liver disease transforming into liver cirrhosis. After 2 weeks, our patient was discharged. However, within 3 weeks after discharge, our patient was rehospitalized for a relapse of acute liver failure, urinary tract infection, and influenza. Leflunomide and methotrexate were not reintroduced before or thereafter. Over a period of 11 months after discharge, her thrombotic microangiopathy subsided, and her renal and liver function fully recovered.

**Conclusions:**

Under a combination of leflunomide and methotrexate, liver toxicity and, for the first time, thrombotic microangiopathy occurred as side effects. Non-alcoholic fatty liver disease may have predisposed for the drug-induced liver toxicity.

## Background

Treatment of active rheumatoid arthritis may necessitate a methotrexate mono- or combination therapy. A combination of methotrexate and leflunomide is regarded as a safe option. However, in 10–20% of patients being treated with this combination therapy, elevated plasma transaminases were documented within 1 year of treatment [1, 2]. Both immunosuppressive drugs are metabolized in the liver. A771726, the active metabolite of leflunomide, has a half-life of up to 2 weeks [3]. The antimetabolite methotrexate competitively inhibits dihydrofolate reductase. Unless taken care of by a folic- or folinic-acid rescue therapy, the resulting folate deficiency may increase the formation of methotrexate metabolites involved in liver toxicity [4].

Here, we present a case of a patient with severe liver toxicity complicated by a thrombotic microangiopathy (TMA) under combination therapy of leflunomide and methotrexate.

## Case presentation

A 63-year-old female patient of Caucasian ethnicity complained of progressive muscle weakness for the past 2 months, sinus tachycardia, dyspnea, petechial skin bleeding of the lower legs, livedo racemosa, yet peripheral edema and ascites. She was transferred from a regional hospital to a third-referral clinic for suspected nephrotic syndrome with hypoalbuminemia and dipstick-positive proteinuria. She was awake, fully oriented, had no neurological symptoms. Her known comorbidities included a metabolic syndrome with obesity (her height was 1.62 m, weight 93 kg, and body mass index 35.4 kg/m^2^), arterial hypertension, type-2 diabetes without oral antidiabetics or necessity for insulin, and dyslipidemia. In addition, a seronegative rheumatoid arthritis had been diagnosed 9 years prior to hospitalization. Medical treatment included methotrexate with a weekly dose of 15 mg, followed by folic acid (5 mg) on the day after the methotrexate administration for 8 years, and leflunomide with a daily dose of 20 mg for 6 years. Concomitant medication consisted of furosemide (40 mg once daily [QD] for 4 weeks), pantoprazole (40 mg twice daily [BID]) for a suspected chronic gastritis, simvastatin (20 mg QD) for hyperlipoproteinemia and metamizole as a pain relief, if needed.

Laboratory work-up showed an oliguric, acute kidney injury (AKI) (stage 1, Acute Kidney Injury Network [AKIN] classification) and acute liver failure (Table 1). In addition, on admission, laboratory test results showed a combined respiratory and metabolic alcalosis with a pH of 7.607 (normal range 7.35–7.45), pCO2 of 3.73 kPa (normal range: 4.27–6.0 kPa), and serum bicarbonate of 28.2 mmol/l (normal range: 22–26 mmol/l). In addition, severe hypokalemia (2.4 mmol/l) due to hypovolemia and alcalosis. A multifactorial anemia due to folate deficiency (plasma level: 5.87 nmol/l, normal range: > 6.8) and hemolysis with the presence of schistocytes was found. The direct and indirect Coombs test and erythrocyte-autoantibody assays (polyspecific, monospecific, cold agglutinins, complement C3d) were negative. Her vitamin B12 plasma levels (809 pmol/l, normal range: 133–675), and D-dimer (6.86 mg/IFEU, normal < 0.5) were elevated. The schistocyte-positive hemolytic anemia with elevated lactate dehydrogenase accompanied by both AKI and thrombocytopenia proved the diagnosis of TMA. Her leflunomide serum level was elevated (120 μg/ml, reference value under therapy: 63 + − 36 μg/ml, Amedes Laboratory, Göttingen, Germany). Plasma activity of ADAMTS13 (a disintegrin and metalloproteinase with a thrombospondin type 1 motif, member 13), a Von Willebrand factor-degrading protease, was decreased to 35% (modified FRETS test, Technoclone, Vienna, Austria). Likewise, ADAMTS13 concentration was decreased to 0.3 μg/ml (normal range: 0.5–1.6 μg/ml).

**Table 1:** Laboratory test results over the hospital stay and at follow-up examinations in an outpatient clinic

Laboratory parameter (unit)	Normal range	Day −1 at primary hospital	Day 1 at referral clinic	Day 2	Day 3	Day 7	Day 9	Day 14, dis-charge	Day 37	11 months later
Sodium (mmol/l)	136–146	132	133	135	139	141	145	143	138	137
Potassium (mmol/l)	3.4–4.5	2.9	2.6	2.4	2.4	4.0	3.8	4.2	2.8	4.2
Calcium (mmol/l)	2.2–2.7	NA	1.7	NA	1.7	2.0	1.9	2.1	1.9	2.4
Creatinine (μmol/l)	< 66	108	87	94	67	62	NA	60	60	77
Urea (mmol/l)	3.6–8.0	NA	9.1	8.8	5.5	4.7	NA	2.2	2.5	NA
Cystatin C (mg/l)	0.5–1.0	NA	2.2	2.1	NA	NA	NA	NA	NA	1.6
Lactate dehydrogenase (μmol/l*s)	< 4.12	19.5	9.8	NA	8.2	7.3	7.4	6.8	5.6	NA
Haptoglobin (g/l)	0.3–2.0	NA	< 0.01	NA	NA	NA	NA	NA	NA	NA
Total bilirubin	< 17	19.5	22.0	NA	21.6	NA	NA	NA	23.0	NA
Total serum protein (g/l)	65–85	37.1	34.0	NA	NA	NA	NA	NA	42	81
Serum albumin (g/l)	35–52	NA	17	NA	NA	NA	25	24	21	43
AST (μmol/l*s)	< 0.52	NA	1.3	NA	NA	0.7	NA	0.6	0.8	0.2
ALT (μmol/l*s)	< 0.56	NA	0.7	NA	NA	0.6	NA	0.5	0.2	0.3
gGT (μmol/l*s)	< 0.63	NA	2.1	NA	NA	2.4		2.3	1.0	2.4
C-reactive protein (mg/l)	< 5	25.5	27.9	NA	NA	10.9	6.5	11.0	21.3	< 1.0
Complement 3 (g/l)	0.9–1.8	NA	NA	0.6	NA	NA	0.9	NA	NA	NA
Complement 4 (g/l)	0.1–0.4	NA	NA	0.2	NA	NA	0.2	NA	NA	NA
Leukocyte count (Gpt/l)	3.8–9.8	NA	NA	NA	NA	6.7	7.4	5.6	7.9	7.0
Platelet count (Gpt/l)	140–440	NA	97	107	101	94	97	100	96	182
Hemoglobin (mmol/l	7.4–9.9	NA	6.9	6.2	5.6	6.2	6.2	6.4	7.6	9.3
International normalized ratio	0.9–1.2	NA	1.9	2.0	1.6	1.7	1.9	1.4	2.2	NA

*ALT* alanin-aminotransferase, *AST* aspartate-aminotransferase, *gGT* gamma-glutamyltransferase, *NA* not applicable

As for the classification of TMA, an enteric infection as a cause of Shiga toxin-producing Escherichia coli associated hemolytic uremic syndrome was not present. Her blood pressure was normal, there was no clinical or laboratory sign of sepsis, thus excluding other forms of secondary TMA. To exclude a cancer-related TMA or an autoimmune disease-related, secondary thrombotic thromboyctopenic purpura (TTP), further diagnostics were performed. Serum protein electrophoresis was unrevealing, except for an elevated alpha-2 globulin fraction (8.8%, normal range: 2.9–4.9%). Acute hepatitis A, B, and C were ruled out. Serum complement 3 was decreased (0.64 g/l), complement 4 was within normal range on admission. Antistreptolysin titer and an autoimmune disease panel (anti-antimitochondrial, -liver-kidney membrane, -antinucleic acid, and -proteinase-3, -myeloperoxide, -glomerular-basement membrane, -anti-double-strand deoxyribonucleic acid (DNA) autoantibodies) results were all negative. The ADAMTS13 inhibitor test result was negative (10 units/ml, cutoff for positivity: 16 units/ml), thereby excluding TTP.

As a main finding, hypoalbuminemia and coagulopathy were found, her liver transaminases were slightly elevated with a higher aspartate aminotransferase (AST) level indicative for liver mitochondrial injury. Her serum ferritin was repetitively found to be high (762 and 674 ng/ml, normal range: 28–365 ng/ml) along with a high transferrin saturation (77.4%) indicative for hemochromatosis. However, the general practitioner had prescribed iron supplements prior to hospitalization. One month later, her transferrin saturation was 40.2%, thereby rendering the diagnosis of hemochromatosis rather unlikely. Alpha-1-antitrypsin and coeruloplasmin were within normal range. The electrocardiogram on admission revealed sinus tachycardia and a peripheral low voltage. Sonography revealed ascites, hepatosplenomegaly, and cholecystolithiasis. Her kidneys were found to be normal in size, with no sign of a post-renal cause of AKI.

For suspected liver toxicity and suspected drug-induced TMA (DITMA), the possible offending drugs, leflunomide and methotrexate, were discontinued. To counteract a bilioenteric cycle, both oral lactulose and cholestyramine (24 g/d) were given from day 2 until discharge. Oral vitamin K was administered to improve the compromised coagulation, 4 units of fresh-frozen plasma and human albumin (100 ml, 20%) were given intravenously from day 2 for acute liver failure. In addition, potassium was given (40 mval/d in 1 l crystalloid solution per day intravenously [IV] for 2 days, and oral potassium supplements 48 mg/d during the hospital stay until the hypokalemia was resolved). In addition, acetazolamid was given for 2 days to counteract the refractory metabolic alcalosis, but without any effect. Blood transfusions were not deemed necessary. Until the differential diagnosis of TTP was excluded, prednisolone therapy was given (60 mg/d for 5 days), then tapered off. During this steroid therapy, one relevant hyperglycemic and iatrogenic hypoglycemic episode occurred. However, after steroid therapy was discontinued, antidiabetic treatment consisted of diet. Repetitive measurements of glycated hemoglobin A1c were below 6.5%. A concomitant urinary tract infection with *Escherichia coli* was treated with cotrimoxazole over 4 days according to the antibiogram. Once the hypoalbuminemia improved, diuresis resumed. On day 5 in hospital, torasemide 10 mg QD was started, and furosemide was discontinued. Her kidney and liver function along with her plasma complement 3, lactate dehydrogenase, and hemoglobin levels all normalized by discharge. By discharge, her body weight had decreased by 9 kg due to the resolution of the edema and ascites. An oral supplemental nutrition for suspected malnutrition-associated hypoalbuminemia was started shortly before discharge. Liver biopsy revealed a non-alcoholic fatty liver disease transforming into liver cirrhosis.

Three weeks after discharge (day 37 after her initial admission), our patient was readmitted for acute liver failure with ascites and edema. Similar to the index hospitalization, severe metabolic alcalosis, hypokalemia, and a urinary tract infection caused by *Escherichia coli*, yet no TMA, were found. Oral ciprofloxacin (500 mg BID for 3 days) was administered according to the antibiogram. After reinitiation of cholestyramine and lactulose, sequential 20% human albumin substitution (5 × 100 ml IV), oral potassium substitution, torasemide dosage increase, and after initiation of the mineralocorticoid antagonist spironolactone (50 mg BID), her acute liver failure improved. Anasarca improved, and our patient lost 20 kg of edema-related body weight. For a hospital-acquired influenza pneumonia (serotype A/H1N1), oral oseltamivir (75 mg BID) was prescribed. At discharge after 4 weeks, a urinary dipstick test was negative for leukocytes and proteinuria. Over a follow-up period of 11 months, our patient fully recovered, the suspected DITMA subsided. In the annual follow-up examinations over 4 years, her kidney and liver function remained stable (data not shown). In view of the overall improvement after drug discontinuation, the observed TMA was classified as a possible DITMA. Both in and out of hospital, a reinitiation of methotrexate and leflunomide was not attempted. The medical treatment of rheumatoid arthritis was switched to nonsteroidal anti-inflammatory drugs. A possible DITMA was reported to the authorities with regard to this case.

## Discussion and conclusions

To the best of our knowledge, we describe a leflunomide- and/or methotrexate-associated DITMA for the first time [6]. As a likely scenario, methotrexate exerted a liver toxicity in the face of folate deficiency [4], thereby prolonging the half-life of leflunomide, as proven by an increased plasma level.

A leflunomide-induced hepatitis [7] was ruled out by liver biopsy. As a consequence, a DITMA, most likely due to an overdosage of leflunomide, occurred. Discontinuation of both methotrexate and leflunomide and leflunmoide elimination by cholestryramine led to an improvement of both liver toxicity and DITMA. Leflunomide has been linked previously to pancytopenia with schistocyte-positive hemolytic anemia, although a concomitant TMA, e.g. due to cobalamine deficiency and/or leflunomide, was not considered there [5].

In the present case, congenital TMA, such as an ADAMTS13 polymorphism, was not ruled out as a reason for her recurrent TMA. However, our patient had no sign of TMA prior or after the index hospitalization. A urinary tract infection was treated with antibiotic therapy on two occasions during the course of the newly diagnosed TMA and 3 weeks afterward. This treatment showed no effect on lactate dehydrogenase levels or on platelet count as TMA-related laboratory parameters. Thus, the urinary tract infection cannot be linked to the TMA observed in the present case. Likewise, sepsis was excluded. The initial elevation of D-dimer was deemed unrelated to the observed TMA, most likely due to hemotoma. A venous thrombosis and/or pulmonary embolism appears to be unlikely in face of the compromised coagulation.

Refractory combined respiratory and metabolic alcalosis with severe hypokalemia occurred, a life-threatening condition. An interstitial pulmonary edema on admission may have accounted for the respiratory component of alcalosis. Loop diuretic therapy resolved both interstitial edema and ascites by discharge, and normalized the respiratory alcalosis component. The metabolic alcalosis component was likely due to intravascular hypovolemia, as clinically shown by the livedo racemosa. The correction of hypovolemia by intravenous gavage of human albumin and human plasma affected both kidney function and the metabolic alcalosis component. A prerenal cause of oliguric AKI is unlikely, as for the presence of edema and for the weight loss achieved by loop-diuretic therapy. Again, the hypokalemia is considered as a consequence of both metabolic and respiratory alcalosis. Both the absence of a pre- or post-renal cause of AKI and the course of the disease over 11 months after drug discontinuation suggest the presence of DITMA. Both the hemolytic anemia and thrombocytopenia resolved over 11 months. The therapeutic measures, i.e. early discontinuation of leflunomide and methotrexate as well as cholestyramine-induced leflunomide elimination showed a good, however, slow treatment response. Except for fresh-frozen plasma, which is known to increase ADAMTS13 activity [8], no escalation therapy such as an anti-complement-directed treatment was performed. As there is no pathophysiological role for total plasma exchange in DITMA, the therapeutic success solely relies on a timely diagnosis and discontinuation of the suspected, offending drugs leflunomide and/or methotrexate. The diagnosis of DITMA is possible, as follow-up data after discontinuation of this combination therapy demonstrated a full recovery. A re-exposure to either immunosuppressive therapy was not performed.

In summary, we present a case of possible DITMA with hemolytic, schistocyte-positive anemia, thrombocytopenia and oliguric, acute kidney failure under the combination therapy of leflunomide and methotrexate. The acute liver failure, in turn, has been facilitated by existing non-alcoholic fatty liver disease transforming into liver cirrhosis, and by a ongoing methotrexate therapy in the face of a folate deficiency. The timely diagnosis of TMA and the consideration of DITMA appear to be crucial for outcome. Rheumatologists and nephrologists should consider the possibility of this rare side effect. In addition, if a DITMA under leflunomide or methotrexate occurs, the use of therapeutic anti-complement strategies should be considered [9].

## Data Availability

The data sets used and/or analyzed during the current study are available from the corresponding author on reasonable request.

## Abbreviations

ADAMTS13: a disintegrin and metalloproteinase with a thrombospondin type 1 motif, member 13
AKI: Acute kidney injury
AKIN: Acute Kidney Injury Network
BID: Twice daily
DITMA: Drug-induced thrombotic microangiopathy
IV: Intravenously
NA: Not applicable
QD: Once daily
TMA: Thrombotic microangiopathy
TTP: Thrombotic thrombocytopenic purpura
